# GPER: An Estrogen Receptor Key in Metastasis and Tumoral Microenvironments

**DOI:** 10.3390/ijms241914993

**Published:** 2023-10-08

**Authors:** Ana Carolina Tirado-Garibay, Elba Andrea Falcón-Ruiz, Alejandra Ochoa-Zarzosa, Joel E. López-Meza

**Affiliations:** Centro Multidisciplinario de Estudios en Biotecnología—FMVZ, Universidad Michoacana de San Nicolás de Hidalgo, Morelia 58893, Mexico; 1050629h@umich.mx (A.C.T.-G.); 1919533f@umich.mx (E.A.F.-R.); alejandra.ochoa@umich.mx (A.O.-Z.)

**Keywords:** GPER, epithelium–mesenchymal transition, metastasis, tumor microenvironment, therapeutic target

## Abstract

Estrogens and their role in cancer are well-studied, and some cancer types are classified in terms of their response to them. In recent years, a G protein-coupled estrogen receptor (GPER) has been described with relevance in cancer. GPER is a pleiotropic receptor with tissue-specific activity; in normal tissues, its activation is related to correct development and homeostasis, while in cancer cells, it can be pro- or anti-tumorigenic. Also, GPER replaces estrogen responsiveness in estrogen receptor alpha (ERα)-lacking cancer cell lines. One of the most outstanding activities of GPER is its role in epithelial–mesenchymal transition (EMT), which is relevant for metastasis development. In addition, the presence of this receptor in tumor microenvironment cells contributes to the phenotypic plasticity required for the dissemination and maintenance of tumors. These characteristics suggest that GPER could be a promising therapeutic target for regulating cancer development. This review focuses on the role of GPER in EMT in tumorigenic and associated cells, highlighting its role in relation to the main hallmarks of cancer and possible therapeutic options.

## 1. The G Protein-Coupled Estrogen Receptor

In the late 1990s, a novel gene was identified in Burkitt’s lymphoma and breast cancer cells that encoded a heptahelical receptor that seemed to be associated with estrogen receptor alpha (ERα). This sequence showed homology to G protein-coupled receptors (GPCRs) and was named GPCR-Br [1,2]. Further, this novel receptor was identified in animal models such as rats, where the homologous gene was detected in cells lacking ERα. These cells responded to 17β-estradiol (E2) stimulation via activating the mitogen-activated protein kinases (MAPK) pathway, and the receptor was named ER-X or GPR41 [3,4]. Nowadays, this receptor is known as G protein-coupled estrogen receptor 1 (GPER/GPR30) and has been identified as an alternative receptor for ERα that triggers cytoplasmic estrogen-signaling and promotes estrogen gene expression [5,6,7,8].

**Figure 1:**
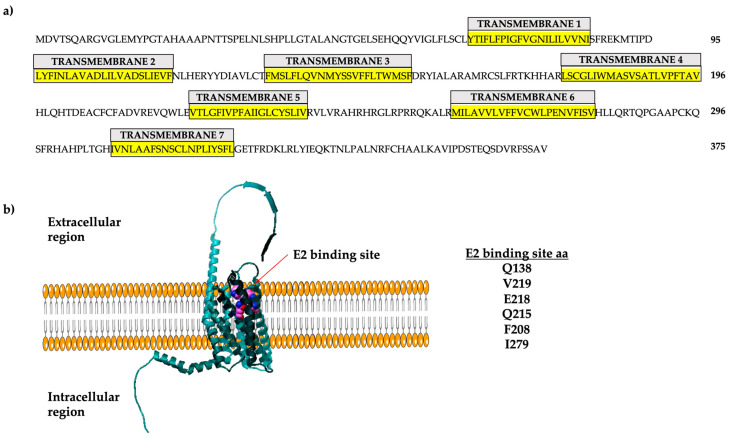
GPER localization and amino acid sequence. (**a**) The sequence of the 375 amino acids of the human protein is shown here. The seven transmembrane regions are indicated in yellow. (**b**) Three-dimensional representation of GPER. The modeling was performed using Swiss Model with the template Q99527.1.A. Modified from [9].

The human GPER gene is intronless, located on chromosome 7, region p22, and encodes a 375 amino acid protein [9]. The GPER promoter region has estrogen response elements (EREs), which allow the binding of estrogen receptors to promote its transcription; consequently, E2 stimulates GPER expression [10]. GPER is a plasma membrane receptor (Figure 1) but can be internalized via a stimulus (e.g., E2) and localized in membranous organelles such as the endoplasmic reticulum and Golgi apparatus [11,12,13,14,15]. GPER has seven transmembrane domains with extracellular N-terminal region. In contrast, the C-terminal region is cytoplasmic and interacts with hydrolases of nucleotide guanosine triphosphate (GTPases) or trimeric G-protein [1,16,17]. GPER can be activated via E2 and inhibitors of ERα, such as tamoxifen (TMX), fulvestrant (ICI 182,780) (FVT), raloxifene (RLX), synthetic ligands (e.g., G1), aldosterone, and natural compounds such as kaempferol [18,19,20,21,22]. These ligands activate GPER to trigger tissue-specific signaling. Some of these ligands are included in the therapeutic scheme against estrogen response cancer, which could explain the re-incidence or aggressiveness of cancer after treating patients.

## 2. GPER/GPR30 Signaling in Normal Tissues

**Figure 2:**
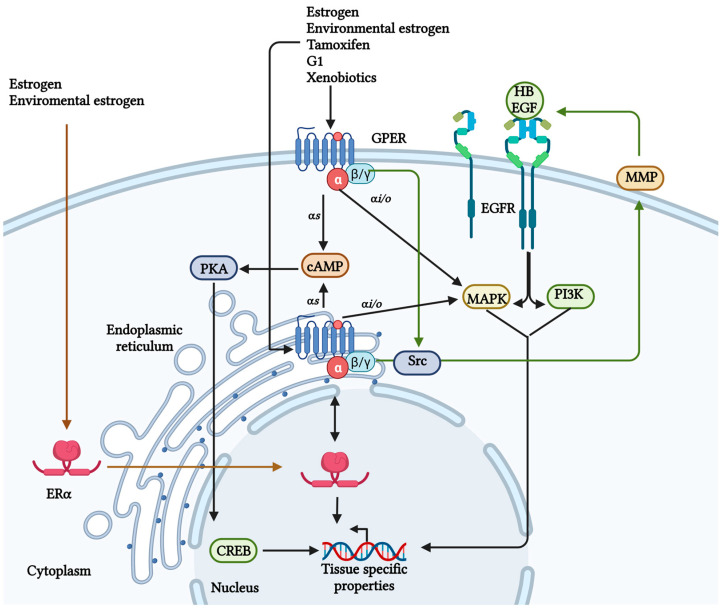
GPER signaling. Different ligands can activate GPER; some are ERα ligands. Once GPER is activated, the G-protein trimer is separated into two subunits, the Gα is related to cAMP and calcium regulation, and the Gβγ can transactivate the EGFR and its signaling. However, a relationship exists between ERα and GPER, favoring the expression of genes related to cancer development [11,30,40]. Created with BioRender.com (accessed on 25 August 2023).

In humans, there are four different classes of G-protein alpha subunit (Gαs, Gαi/o, Gαq/11, and Gα12/13). It has been reported that GPER can be coupled to a Gα type s (Gαs) or Gα type i/o (Gαi/o); thereby, activating different routes of signaling and regulation [6,23,24,25]. Once GPER is activated, the G-protein trimer is separated into two subunits, Gαs and Gβγ (Figure 2). The Gαs subunit activates adenylate cyclase and cyclic adenosine monophosphate (cAMP) production to mobilize intracellular calcium (Ca^2+^). Moreover, GPER decreases plasma membrane Ca^2+^-ATPase (PMCA) activity, maintaining Ca^2+^ in the cytoplasm and representing a secondary Ca^2+^ regulation mechanism [11,18,26,27]. This Ca^2+^ regulation improves brain development, neuronal growth, and communication [28], and also stimulates potassium (K^+^) efflux, which promotes nitric oxide (NO) production in the endothelium, resulting in a vasodilator effect [25,29].

On the other hand, GPER also seems to be associated with a Gαi/o because the pertussis toxin inhibits the GPER-mediated ERK ½ signaling [25]. Also, the Gβγ subunit could transactivate the epidermal growth factor receptor (EGFR) via heparin-binding EGF-like growth factor (HB-EGF) activating the phosphatidylinositol 3-kinase (PI3K) and MAPK pathways (Figure 2) [30,31]. Furthermore, these mechanisms have been associated with the proliferation of non-tumorigenic breast cells MCF10A ex vivo [32], dendritic spin density related to spatial learning and memory [33,34,35], and glucose and lipid homeostasis [36].

The binding affinity of E2 to GPER is 3–6 nM, whereas ERα’s is 0.1–1 nM. It is noteworthy that there has been no reported activation of GPER via E2 in epithelial and hippocampus models in vivo [37]. However, other ligands, such as G1, activate the receptor [38], showing the importance of using different study models. Nevertheless, as with other GPCRs which can be allosterically regulated, a GPER ligand-independent activity related to a domain involved in protein–protein interactions in the C-terminal, called PDZ motif, has been reported. This activation is associated with proliferation, migration, and invasion [8,25,39]. These characteristics are related to neoplasia development, which suggests that GPER could be a cancer-relevant regulator.

## 3. GPER Expression in Cancer

GPER exhibits a tissue-specific activity which can be pro- or anti-tumoral, depending on the cancer type. The receptor level varies depending on its role; when it shows anti-tumoral activity (e.g., gastric, ovarian, and hepatic cancer), its expression is lower than in normal tissues. Research groups have reported that its activation or overexpression in these cancer types is related to a decrease in cancerous properties, supporting the anti-tumoral role of GPER in these cancer types (Table 1) [31,41,42]. An example of this anti-tumoral activity is that GPER activation inhibits the growth of malignant cells through GPER/ERK signaling in hepatocarcinoma and xenograft models [43]. Additionally, the low expression of this receptor in the hepatocarcinoma model has been related to promoter methylation and histone H3 deacetylation [44]. Likewise, GPER anti-carcinogenic effects have been reported in prostate cells and xenografts involving G2 cell-cycle arrest [45]. Moreover, the anti-proliferative activity of GPER has been observed in ovarian cancer [46] and has been related to epigenetic regulation, such as the H3K4me3 mark [41]. Furthermore, the GPER activation via tamoxifen inhibits proliferation, migration, and invasion in pancreatic cancer and regulates the tumorigenic environment, decreasing the anti-inflammatory macrophage phenotype M2 [47].

However, there are some cancer types where GPER plays a pro-tumorigenic role (Table 1). Although intracellular GPER is mainly located on the endoplasmic reticulum, its cytoplasmic location has been related to the advanced cancer stage [27,48]. A higher GPER expression in cancer cells and tissues favors cancer development, and GPER levels serve as indicators for survival prognoses, where elevated levels are related to bad outcomes. Some examples of the above have been observed in lung, prostate, cervical, glioblastoma, and breast cancer models [49,50,51,52,53]. Moreover, GPER expression is related to stemness features in breast cancer stem cells (BCSC) through phosphorylation of BCL2-associated agonist of cell death (BAD) promoting cell survival, but the silencing of GPER results in a decrease in BCSC in vivo [54,55]. However, in breast cancer cells, GPER is related to the stabilization of the actin cytoskeleton and the up-regulation of Yes-associated protein 1 (YAP) and transcriptional coactivator with a PDZ-binding domain (TAZ) via the activation of Gαq-11, phospholipase C beta (PLCβ)/PKC and Rho/Rho-associated protein kinase (ROCK) signaling [56]. In tumorigenic cells, GPER activation has been related to the expression of cell adhesion molecules (CAMs), metalloproteases (MMPs), focal adhesion (FA) pathway genes, cyclin D1 (CCND1), anti-apoptotic protein B cell lymphoma (Bcl-2), and transcription factors such as c-fos [57,58,59]. Some ERα-target genes demonstrate that GPER can promote the expression of ERE genes as an ERα-alternative receptor [60,61]. These proteins are related to carcinogenic characteristics such as proliferation, energetic deregulation, metastasis, and immune and apoptotic evasion [62], which indicate that GPER is a pro-carcinogenic regulator. However, a dual GPER activity in testicular cancer has been reported. The germ neoplasm group is related to high receptor levels, and a GPER E2-activation induces proliferation through the ERK 1/2 pathway in vitro. On the other hand, GPER activation induces apoptosis in the Leydig cell tumors, showing a specific activity of the receptor upon the type of testicular cancer [63].

**Table 1:** Role of GPER in different cancer types.

Anti-Tumorigenic Role of GPER			
Cancer Type	Receptor State	Activity	Mechanism
Hepatic	Overexpression	Anti-tumoral	Through GPER in hepatocarcinoma [43]
Prostate	Low expression	Anti-tumoral	G2 cell-cycle arrest [45]
Ovarian	Overexpression	Anti-tumoral	Epigenetic regulation, such as the H34me3 mark [42]
Pancreatic	Activation by tamoxifen	Inhibits the proliferation, migration, and invasion process	Regulates the tumorigenic environment, decreasing the anti-inflammatory macrophage phenotype M2 [47]
Leydig cell tumors	Low expression	Anti-tumoral	Induces apoptosis [63]
**Pro-Tumorigenic Role of GPER Receptor**			
**Cancer Type**	**Receptor State**	**Activity**	**Mechanism**
Lung	Overexpression	Pro-tumoral, poor prognosis	The expression of GPER on the cell surface is related to cell survival [49]
Cervical	Overexpression	Pro-tumoral	Induction of tumor-promoting claudin-1 [50]
Prostate	Stimulation by estrogen	Increased stromal components and prostatic fibrosis accelerate the clinical progression	GPER activates EGFR/ERK and P13k/AKT HIF1α pathways [51]
ER-positive breast tumor	Overexpression	Poor prognosis	ERK1/2 pathway [52]
Triple-negative breast cancer	GPER interacts with FAK	Increases the migration, adhesion, and invasion	Increasing focal adhesion points [52]
Glioblastoma	Activation of GPER	Promotes tumor growth	Induces the epidermal growth factor receptor, triggering the ERK pathway [53]
Testicular germ neoplasms	Overexpression	Promotes proliferation	GPER activates the ERK 1/2 pathway [63]

Another reported example of GPER dual activity occurs in colorectal cancer and depends on gender. In a patient study, men with higher GPER levels had a better prognostic outcome; meanwhile, women showed the opposite effect [64]. In contrast, in vitro studies have demonstrated that GPER activation is related to colorectal cancer, favoring proliferation and migration [65].

On the other hand, the GPER-induced gene expression and the receptor can be epigenetically regulated. For example, a negative relationship between miR-338-3p/miR-124 and GPER has been reported. miR-338 inhibits c-fos translation, and miR-124 targets CD151, decreasing proliferation in hepatocarcinoma and breast cancer, respectively. Nevertheless, the E2-activation of GPER diminishes miR-338-3p/miR-124 expression (Table 2) [66,67]. On the other hand, there is a negative relationship between miR-148a and GPER, where receptor activation increases cell proliferation and inhibits apoptosis. However, antagonists of GPER, such as G15, up-regulate miR-148a and promote cell death in ovarian endometriosis [68] and breast cancer [56]. In the latter, it has been reported that down-regulation of miR-148a is related to an increase in CD151, HOX transcript antisense RNA (HOTAIR), and histocompatibility antigen class I, G (HLA-G) levels, favoring the immune system evasion [56]. Furthermore, an epigenetic regulation of GPER expression in endometrial carcinoma has been reported because miR-424 targets GPER, inhibiting E2-induced proliferation [69]. However, GPER also acquires relevance in the carcinogenic process because it seems critical in metastasis development in some cancer types.

## 4. Epithelial–Mesenchymal Transition and Metastasis Development

**Figure 3:**
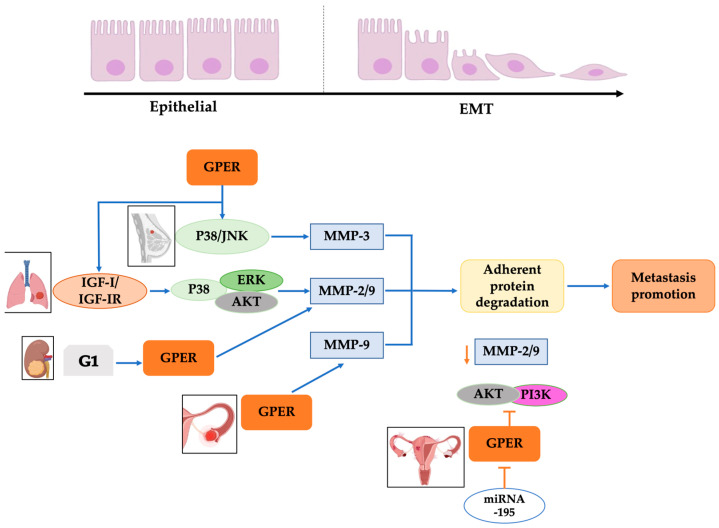
GPER’s relationship with EMT in cancer. Overexpression of GPER in breast, lung, kidney, and ovarian cancer cells generates a kinase activation pathway that stimulates MMP-3, MMP-2/9, and MMP-9 activation, which degrades the adhesive proteins, favoring the promotion of metastasis. Additionally, specific miRNAs, like miRNA195, block GPER activation in ovarian cancer cells; consequently, MMP-2/9 activation via AKT/PI3K is diminished, preventing the degradation of adhesive proteins and interrupting the promotion of metastasis. Modified from references [31,49,52]. Created with BioRender.com (accessed on 25 August 2023).

Metastasis is the dissemination of tumorigenic cells to other organs, allowing cancer cells to invade them and initiate a new tumor. This process requires phenotype plasticity to dedifferentiate mature cells back to progenitor states through the loss of linkage proteins and gain of mobilization phenotype, a phenomenon called epithelial–mesenchymal transition (EMT) (Figure 3) [62]. GPER has a relevant role in EMT because it promotes metalloprotease expression through the p38/JNK pathway, favoring the degradation of adherent proteins such as E-cadherin and acquiring a migratory phenotype in several types of cancer, such as breast, lung, colon, and ovarian [31].

GPER levels are directly related to lung carcinoma status. Higher levels are associated with EMT through MMP-2/9 expression via insulin-like growth factor-I (IGF-I)/IGF-IR by ERK, p38, and AKT pathways [72]. The activation of GPER via G1 also promotes the expression MMP-2/9 in renal carcinoma in vitro, improving the acquisition of a metastatic phenotype [73]. However, as mentioned, GPER levels can be regulated via micro RNAs (Table 2), and miR-195 targets the mRNA of the receptor, inhibiting the PI3K/AKT-induced MMP-2/9 expression in endometrial carcinoma; therefore, avoiding the EMT [73]. Additionally, E2-activation of GPER is related to the down-regulation of miR-124, allowing the metastasis progression in breast cancer [67]. Nevertheless, although this carcinoma has a low receptor level, GPER activation is related to MMP-9 expression and metastasis development in ovarian cancer [39] (Figure 3).

Moreover, GPER activation is related to FAK activation in triple-negative breast cancer (TNBC) and triggers ERK1/2/AKT signaling, promoting metastatic characteristics, such as migration and invasion [74]. In the same way, the formation of tubes via endothelium cells exposed to a conditioned medium from GPER-activated TNBC is related to the vessel formation required for migration and tumor sustaining [75]. Indeed, GPER antagonists, such as G15, have demonstrated inhibition of EMT in TNBC through sensitization to doxorubicin, up-regulation of E-cadherin, and down-regulation of vimentin [76]. These changes in expression can be associated with a network between Notch1, hypoxia-inducible factor 1α (HIF-1α), and GPER that improves EMT through increasing HIF-1α recruitment to the Snail promoter via nuclear GPER [77]. Snail is a zinc-finger transcriptional factor, and its expression is related to the deacetylation of the E-cadherin gene promoter (CDH1) and, therefore, the down-regulation of E-cadherin expression [78]. On the other hand, it has been reported that GPER activation inhibits migration and invasion [79] and stimulates miR-199a-3p expression and CD151 down-regulation, inhibiting EMT in TNBC [71]. These reports show contrasting data about GPER’s role in breast cancer. Therefore, more studies are required to elucidate the role of GPER in these events.

## 5. Tumor Microenvironment Favors EMT through GPER

**Figure 4:**
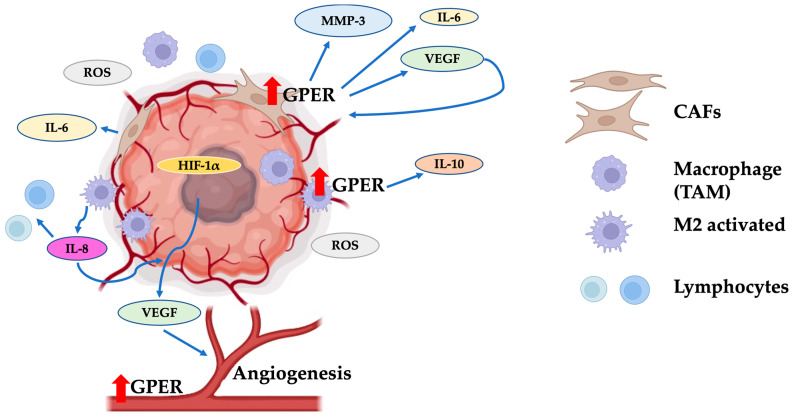
The tumor microenvironment favors EMT through GPER. GPER overexpression in carcinoma-associated fibroblasts (CAFs) increases the production of MMP3, IL-6, and VEGF. These molecules improve tumor viability via inducing blood vessel formation. Additionally, tumor-associated macrophages (TAMs) increase IL-10 production through GPER activation, generating a protective microenvironment around the tumor mass. The expression of GPER on endothelium has been shown to influence the improvement of tumor angiogenesis. CAFs: carcinoma-associated fibroblasts; TAMs: tumor-associated macrophages; M2 activated: activated macrophages of type M2; ROS: reactive oxygen species; HIF-1α: hypoxia-inducible factor 1α; VEGF: vascular endothelial growth factor. Red arrows indicate un increase in the GPER levels. Modified from references [75,77,82,88]. Created with BioRender.com (accessed on 25 August 2023).

As aforementioned, EMT consists of re-programming expression patterns that stimuli of the environment can favor [62]. The tumor microenvironment consists of normal cells, tumor cells, tumor stromal cells, including stromal fibroblasts, endothelial cells, immune cells such as macrophages and lymphocytes, and non-cellular components (Figure 4). These elements are involved in a dynamic and bidirectional interaction between tumor cells and surroundings through secreted soluble molecules responsible for the horizontal information transfer between cellular/non-cellular communicating cells [80].

The carcinoma-associated fibroblasts (CAFs) are normal fibroblasts activated via growth factors (e.g., transforming growth factor β (TGFβ), hepatocyte growth factor, fibroblast growth factor), and transcription factors (e.g., nuclear factor-kappa B (NF-κB) and heat shock factor-1 (HSF-1)) [81]. CAFs are a significant component of the tumor stroma and produce MMP-3 and cytokines that favor EMT [82]. In addition, these cells express GPER, and its activation up-regulates IL-6, vascular endothelial growth factor (VEGF), and connective tissue growth factor (CTGF) [27]. Also, the CAF secretome improves the expression of vimentin, ZEB1, and Snail in cancer cells, which are mesenchymal proteins essential for cancer progression [22]. Consequently, the activation of GPER in CAFs stimulates the secretion of proteins that activate the cancer cells, promoting a re-programming of an epithelial expression pattern to a mesenchymal [83,84]. Furthermore, CAFs promote TMX and FLV resistance in breast cancer and EMT in hepatocarcinoma via IL-6/IL-6R/STAT3 pathway [85,86,87]. However, it has been reported that 3-methylcholanthrene activates the EGFR/ERK/c-Fos signaling in CAFs through an aryl hydrocarbon receptor (AhR)-GPER mechanism favoring the tumor growth via the up-regulation of cyclin D1 and CYP1B1 [56]. Also, GPER has a role in this resistance because the receptor levels are higher in drug-resistant cancer cells. However, when cells are exposed to receptor antagonists, cell sensibility is recovered [14]. This resistance is related to an aggressive phenotype and mesenchymal protein expression, which suggests that GPER promotes drug resistance and cancer progression via EMT development.

Although immune cells must locate and eradicate tumorigenic cells, they can also contribute to the development of tumors. A chronic inflammatory state is a risk factor for cancer development because prolonged exposure to pro-inflammatory cytokines (e.g., TNF-α) and ROS promotes DNA and tissue damage [89]. This inflammatory state favors the development of cancer cells, and the anti-inflammatory cells (M2) surrounding the neoplasm camouflage the tumor from the immune system, preventing the elimination of affected cells and allowing EMT development [82,90]. Several research groups have reported that GPER is expressed in monocytic cells, such as CD14+ monocytes, in vitro differentiated macrophages, and tissue-resident macrophages [40]. Also, GPER activation is related to expanding regulatory T-cell populations and secretion of the anti-inflammatory cytokine IL-10 from M2-polarized cells [20]. Moreover, it has been reported that exosomes of these macrophages could activate the PI3K-AKT pathway in gastric cancer cells and induce EMT. At the same time, tumorigenic cells stimulate M2 polarization, creating a positive feed-loop [91]. In addition, GPER activation via diethylstilbestrol [92], bisphenols A (BPA), bisphenol AF (BPAF) [93], 17α-ethynylestradiol (EE) [94], and zearalenone (ZLN) [95] have been related to monocyte activation through an increase in the receptor for activated C kinase 1 (RACK1) levels in the cell line THP-1. Also, EE, E2, and ZLN favor the production of LPS-induced pro-inflammatory cytokines, such as TNF-α and IL-8, and the up-regulation of CD86 [95]. However, VIN, atrazine (ATZ), and cypermethrin (CYP) decrease these proteins [96]. On the other hand, the EE and VIN inhibit RACK1 in a primary culture of female donors [97], highlighting the role of GPER in innate immune response regulation.

## 6. Role of GPER in Angiogenesis

Angiogenesis involves creating new vessels that dispense nutrients and is a mechanism through which malignant cells can disseminate to other tissues. Endothelial cells are critical for tumor progression because vessel formation contributes to cancer dissemination and maintenance. Estrogens are related to vascular protection through receptors ERα, ERβ, and GPER [98]. They are associated with protection in cardiovascular diseases, mainly in pre-menopausal females [88], diminishing the inflammation and the NO production mediated in endothelial cells [99]. The ERβ shows increased expression after vasculature injury in males and females and has been related to a protective effect [100]. In addition, the estrogens are tightly linked to the angiogenesis process, stimulating the ERs [101]. However, information about GPER’s protective activity in the endothelium is scarce. GPER mediates the activation of transduction pathways in different processes related to tumorigenesis, such as angiogenesis favoring tumor growth [102] or stimulating endothelial cell proliferation [103]. Some studies have shown that the activation of GPER via G1 inhibits the proliferation of endothelial cells, inhibiting DNA synthesis and arresting the cells in the S and G2 phases [98]. Also, E2 and G1 activate the GPER/EGFR/ERK/c-fos signaling pathway and increase VEGF via up-regulation of HIF1α [104], which is directly related to vessel formation. Also, E2 regulates endothelial cell tube formation in injury and has protective effects associated with GPER in endothelial cells [101]. For this reason, GPER has been proposed as a possible therapeutic target to control the growth of some tumors, such as breast cancer.

## 7. Therapeutic Alternatives against Cancer Targeting GPER

Given the pro-tumorigenic role of GPER, it has been proposed as a therapeutic target. There are a variety of GPER antagonists, such as G15, G36, CIMBA, MIBE, PBX1, PBX2, C4PY, and CPT. The abovementioned drugs have been tested in breast cancer and CAF in vitro and in vivo, resulting in a GPER-dependent G1/E2-signaling inhibition [105,106]. Additionally, chimeras made from an E3 ubiquitin ligase ligand called PROTACs degrade the target; in particular, the E2-PROTAC acts against GPER [107]. However, GPER expression is simultaneous with ERα in some estrogen-responsiveness cancers; as a result, anti-estrogenic compounds must be reconsidered because drugs such as TMX and FVS act as a ligand for GPER [20]. As mentioned, CAFs improve EMT; thus, novel therapies have been focused on them, but their use is related to severe secondary effects such as muscle loss and even death [108]. On the other hand, triptolide is a drug reported as impairing the induction of EMT via CAFs in cancer cells. Also, there are novel therapies against signaling pathways related to the development of cancer properties, such as AG490, a drug against the JAK/STAT signaling involved in EMT that can be triggered via GPER activation [109]. These findings suggest that GPER could be a promising therapeutic target that can act at different levels, regulating the cancer cell and its surrounding components.

## 8. Discussion

The role of GPER in cells is beginning to be understood. GPER has been associated with several physiological functions, such as angiogenesis, brain development, and homeostasis [28,101], but it also participates in several pathological processes, such as carcinogenesis and metastasis development [74,75,76]. Although GPER improves the correct development of neuronal, connective, and epithelial tissues [35,36], changes in its expression can deregulate homeostasis and trigger malignant development [31]. GPER expression levels depend on the cancer type, which could serve as a prognosis marker for the tissue affected [49,50,51]. Because the receptor has a pro-tumorigenic role and its expression is related to cancer status, GPER could become a therapeutic target and complete a drug network to enhance a treatment scheme. Likewise, identifying some miRNAs that can inhibit the receptor and downstream proteins represents a novel therapeutic alternative for the treatment of cancer. When the receptor exhibits an anti-tumoral activity, its ligands, downstream proteins, and associated miRNAs should be well studied to determine their potential as therapeutic alternatives.

Metastasis is a challenge for eradicating cancer because the dissemination of carcinogenic cells affects different tissues and could promote an organic failure; moreover, the number of malignant cells increases, and they acquire diverse phenotypes based on the environment in which they are established [110]. The critical step for metastasis development is the EMT, where the cell attachment decreases and the cells become migratory [62]. GPER plays a relevant role in EMT development because it is expressed in malignant and environmental cells [27,40]. Receptor activation triggers changes in the immunological cells that affect the cancer cells, stimulating the expression of mesenchymal proteins and creating a tumorigenic environment. In contrast, the activation of GPER in the malignant cells allows structural and expression changes needed to invade other tissues [85,86,87]. A remarkable point is that GPER promotes monocyte activation and LPS-induced pro-inflammatory cytokines [56], providing a potential strategy to enhance the immune response. Additionally, GPER has multiple ligands, including some of the drugs used for cancer treatment and xenobiotics; thus, representing an advantage for cancer development and a challenge for medical procedures [20,21,92,93,94,95,96,97].

**Figure 5:**
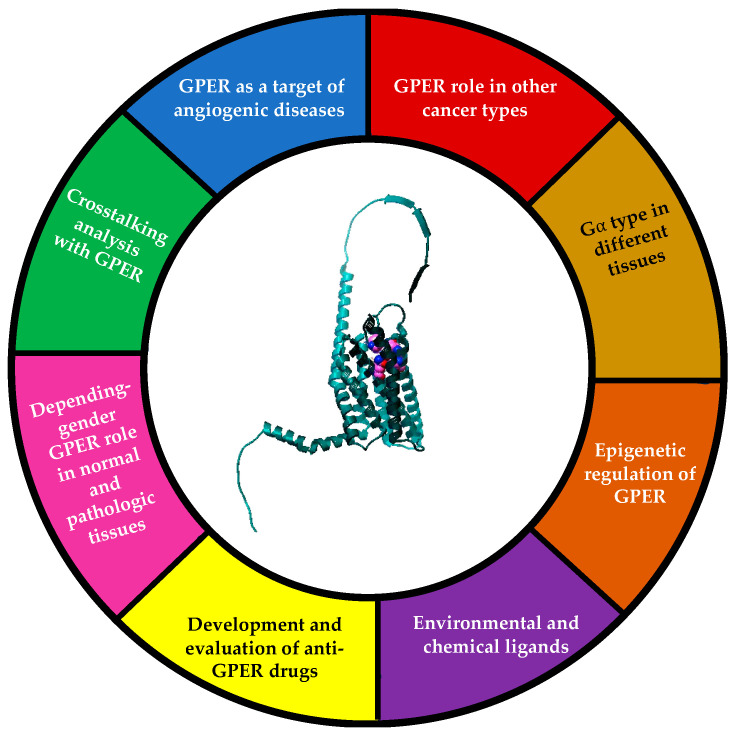
Future trends in GPER study.

Nevertheless, all cancer types have a metastasis pattern, which, if it is not compulsory, is a guide of behavior, and this pattern is based on closeness, vessels, and the function of the tissue. For example, breast cancer usually disseminates to the lung, while prostate cancer frequently invades nodes [111,112]. With regards to GPER tissue-specific activity, it is interesting that cancer types with determinate GPER activity develop metastases to organs where the receptor has the same effect, suggesting that if a cancer type is GPER pro-tumorigenic, its dispersion will affect an organ where the new tumor possibly has the same GPER activity, and a focused therapy against this receptor would control primary and metastatic tumors. Furthermore, identifying this receptor in cancer types without therapeutic targets, such as TNBC [76,77], represents an opportunity to develop new drugs against cancers with poor or incomplete treatment. Moreover, whilst there has been considerable research into GPER, there is still contrasting information. This represents an opportunity for future research to elucidate the tissue-specific and ligand-dependent activity in in vitro, ex vivo, and in vivo models, and come to an understanding of the differences reported through the years. Moreover, it will be interesting to evaluate the ligand-independent signaling of the receptor in cancer where GPER is pro-tumorigenic, and, assess if the use of receptor antagonists is enough to inhibit the carcinogenic activity or if the allosteric activation compensates for the lacking functions (Figure 5).

## 9. Conclusions

GPER has several activities in metastatic development, which justifies proposing this receptor as a therapeutic target to avoid EMT in various cancer types. Also, it is necessary to rethink the current schemes for cancer treatment where GPER ligands are included.

## 10. Future Directions

GPER is a novel receptor that must be well studied both in in vitro and in vivo models to determine its role in normal and pathologic processes. It should also be investigated with regard to whether or not it could be an efficient therapeutic target against cancer that would help current therapy schemes. There have been contradictory reports regarding GPER tissue-specific signaling which must be clarified, and the reasons for these discrepancies must be determined. Moreover, the possible activation via other estrogens, drugs, and estrogen-like ligands must be analyzed to determine the signaling cascades activated (Figure 5).

## Figures and Tables

**Table 2:** Role of miRNAs in GPER signaling in different cancer types.

miRNA	Target	GPER Regulation	Effect
miR338-3p	c-fos	Negative	Proliferation decreases in hepatocarcinoma [66]
miR-124	CD151	Negative	Proliferation decreases in luminal breast cancer [67]
miR148-a	Human leukocyte antigen-G	Negative	Cell death in ovarian endometriosis [68]
miR-424	GPER	Negative	E2-induced proliferation in endometrial carcinoma [69]
miR-195	GPER	Negative	PI3K/AKT-induced MMP-2/9 expression in endometrial carcinoma [70]
miR-199a-3p	CD151	Positive	Promotes EMT in triple-negative breast cancer [71]
